# Identification of Dynamic Behavior Models of Concrete B22.5

**DOI:** 10.3390/ma16062259

**Published:** 2023-03-11

**Authors:** Anatoly M. Bragov, Andrey K. Lomunov, Mikhail E. Gonov, Aleksandr Yu. Konstantinov, Leonid A. Igumnov, Victor A. Eremeyev

**Affiliations:** 1 Research Institute of Mechanics, National Research Lobachevsky State University of Nizhny Novgorod, 603022 Nizhny Novgorod, Russia; 2 Department of Civil and Environmental Engineering and Architecture (DICAAR), University of Cagliari, Via Marengo 2, 09123 Cagliari, Italy

**Keywords:** ultimate stress, concrete, strain rate, Kolsky method, identification, behavior model, fracture energy

## Abstract

We discuss experimental and numerical studies of the deformation and destruction of fine-grained concrete B22.5 under dynamic loading. The experiments were carried out using the Kolsky (or split-Hopkinson pressure bar) method, and its modifications in the strain rate range from 400 to 2000 s^−1^. The rate dependences of ultimate stresses and fracture energy in tension and compression are obtained. Based on experimental data, the identification of the dynamic component of two models from the LS-DYNA computational complex was carried out: *MAT_CONCRETE_DAMAGE and *MAT_CSCM. The results of a comparative analysis of the identified models based on single-element modeling and comparison with experimental data are presented. It is shown that the obtained experimental strain rate dependences of the fracture characteristics can significantly improve the predictive ability of the model compared to the default parameter set. Information about the rate dependence of the fracture energy in *MAT_CSCM model makes it possible to more realistically simulate the behavior of the material beyond the ultimate stress.

## 1. Introduction

For reliable numerical simulation of the behavior of various structures made of concrete and fiber-reinforced concrete under conditions of high-speed impulse loading, the deformation models and failure criteria are needed. These models should adequately describe the behavior of the material in a wide range of strain rates and temperatures under simple and complex loading conditions. These models should also adequately describe the behavior of the material under various types of stress–strain state: uniaxial stress state in compression and tension, uniaxial deformation in compression, shear, and combined state. In addition, it is important to take into account the moisture content of concrete and the temperature at which it is used.

To equip the deformation model and fracture criteria of concrete and fiber-reinforced concrete with the necessary parameters, a complex experimental study of the dynamic deformation of concrete samples under various loading conditions is required. As a result of such experiments, deformation diagrams (strain–stress *σ*(*ε*)) are obtained. Furthermore, the ultimate strength and deformation characteristics of concrete and fiber-reinforced concrete are determined, taking into account the types of tests (compression, tension, shear, complex stress) at various strain rates and stress growth rates. The energy intensity and crack resistance are also evaluated. The dependences of the specified parameters on the strain rate or the rate of stress growth are obtained as a result of data analysis. Based on the obtained parameters of the mechanical properties of concrete and fiber-reinforced concrete, a particular mathematical deformation model is identified. The parameters of failure criteria are also determined.

Then, the mathematical models and fracture criteria of concrete and fiber-reinforced concrete are verified. That is, the adequacy of the model is assessed under conditions that differ from those in which the mechanical properties were obtained to identify the models.

Thus, in order to obtain adequate deformation models and fracture criteria of concrete and fiber-reinforced concrete, a system of basic experiments should be implemented. Based on these results, a certain model of concrete and fiber-reinforced concrete is identified. A system of verification experiments is needful, based on which the adequacy of the identified model and failure criteria are assessed.

The dynamic properties of brittle materials are important in various fields of military, industrial, and civilian activities. Due to the transient nature of the load, dynamic testing of brittle materials is very different from that of the ductile materials. In addition, dynamic tests are more complex than static ones. Dynamic tests are usually carried out according to the Kolsky method using a split-Hopkinson bar. The Kolsky technique is a fairly reliable method for measuring the dynamic properties of brittle materials at high strain rates. This method was proposed by G. Kolsky in 1949 [1,2] to evaluate the dynamic response of various metals under the influence of high loads or strain rates. Shortly thereafter, various researchers began using various modifications of the Kolsky method to test brittle materials, such as concrete, ceramics, and rocks [3]. Several comprehensive reviews have been carried out regarding the dynamic behavior of brittle materials, such as mortar, ceramics, concrete, and rocks [4,5,6,7]. Dynamic experimental methods [8,9,10,11,12] were considered to obtain the properties of brittle materials under various types of stress–strain states. Significant progress has been made regarding the quantification of the various dynamic properties of rock-like materials thanks to advances in split=Hopkinson pressure bar (SHPB) experimental techniques.

In the work, the Kolsky method and its modifications are used to obtain experimental data on the natural laws of high-speed deformation and fracture of fine-grained concrete samples, and several models from the library of the LS-DYNA calculation complex are identified based on this data.

## 2. Materials and Methods

**Figure 1:**
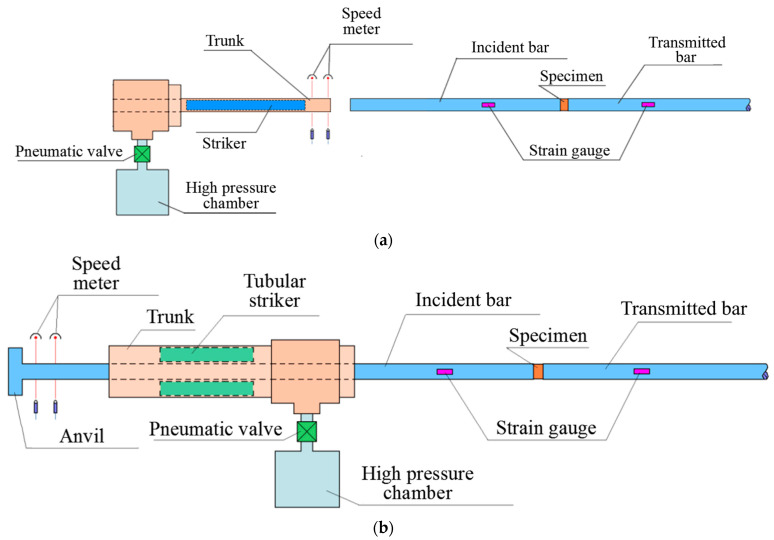
Schemes of test setups: (**a**) for compression, (**b**) for tension.

To test samples under dynamic compression and tension, we used installations that implement the classical split-Hopkinson pressure bar (SHPB) method (Figure 1a) and its modification (Figure 1b) [13]. The histories of changes in strains, strain rates, and stresses in the samples were calculated using the formulas proposed by Kolsky [1,2,13] using the strain pulses recorded in elastic measuring bars. The loading condition (strain rate) was varied by changing the impactor speed. The speed of the impactor depends on the pressure in the chamber of the air gun. It was measured using a light speed meter set.

The experimental setup for direct tension (Figure 1b) [13] differs from the traditional SHPB setup for compression in that the tubular impactor is accelerated in the barrel by a gas gun in the direction opposite to the sample. The tubular impactor hits the anvil, which is fixed at the end of the incident measuring bar, thereby exciting an elastic tension wave in it. The sample is glued either directly to the measuring bars or to special threaded nozzles that are screwed into threaded sockets at the ends of the measuring bars. The processing of experimental data in the split-Hopkinson tension bar (SHTB) setup is carried out using the main dependences of the SHPB method.

**Figure 2:**
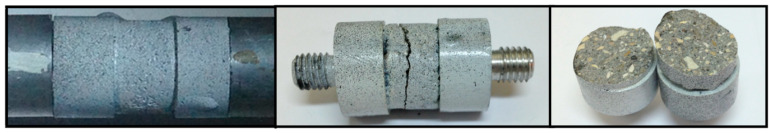
Fixing the sample in a tensile experiment.

Samples for direct tension were glued with epoxy glue with the addition of tungsten powder to increase the strength of the adhesive seam with the replaceable threaded nozzles (Figure 2). Threaded nozzles were screwed into the threaded holes of the measuring bars. The test results showed that the strength of the adhesive joint exceeds the tensile strength of the sample (Figure 2).

For experimental studies according to the SHPB and SHTB methods, both in compression and in tension, cylindrical specimens of a circular cross section are required. The test specimen is placed between the ends of two long coaxial measuring bars. To transfer the shock pulse from the incident bar to the sample and then to the transmitted bar, it is necessary to ensure a tight fit of the test sample to the end of the transmitted bar. In this regard, the Kolsky method imposes a number of high requirements on the samples, such as uniformity of the material and minimum roughness of the end surfaces of the sample. The parallelism of the ends of the sample and their perpendicularity to the axis of the cylinder must necessarily be ensured so that eccentricity does not occur, causing bending of the sample. The optimum ratio of sample diameter to sample length should be between 1 and 2 to reduce the effects of inertia and friction. To reduce the scale effect when using measuring bars with a diameter of 20 to 60 mm, it is desirable to test fine-grained concrete with filler fractions from 1 to 3 mm.

Tests were carried out on samples of fine-grained concrete of class B22.5 obtained via semi-dry vibrocompression. As part of fine-grained concrete, cement grade 500D (consumption 430 kg per 1 m^3^), coarse sand (module 3 mm, consumption 1435 kg per 1 m^3^), fine sand (module 1.6 mm, consumption 355 kg per 1 m^3^), plasticizing Murasan BWA 16 additive (consumption 1.6 kg per 1 m^3^), and water (consumption 120 kg per 1 m^3^) are used.

Concrete workpieces were cut into plates using a Cedima CTS-57-G stone-cutting machine, and specimens of the required size were drilled from the plates using an NS-12M table drilling machine and diamond crowns. Concrete samples for dynamic tests were made with a diameter of 20 mm and a length of 10 mm.

Concrete workpieces for the manufacture of specimens were obtained in the factory. Quasi-static strength, which corresponds to the concrete class B22.5, was provided by the manufacturer.

## 3. Results of an Experimental Study

The test parameters for dynamic compression and tension, as well as some parameters determined from their results, are presented in Table 1 and Table 2.

**Figure 3:**
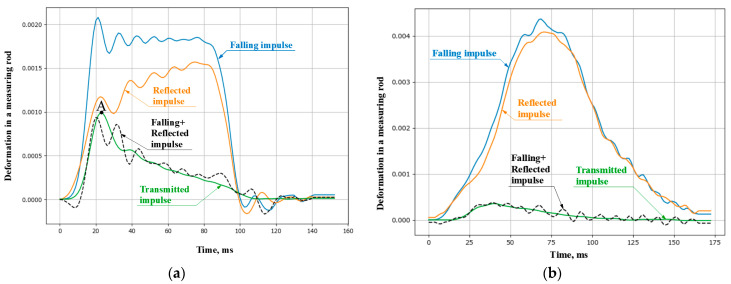
Pulses in measuring bars: (**a**) without an impulse shaper at a strain rate of 1100 1/s and (**b**) with an impulse shaper at a strain rate of 1250 1/s.

Under conditions of dynamic compression, the concrete was tested at seven different speed modes. Five repeated tests were performed for each mode. The strain rates were in the range from 400 to 2000 1/s. To ensure a smooth increase in the load, three modes out of seven were carried out using copper pulse shapers. Figure 3 shows the results of checking the fulfillment of the force equilibrium conditions in the sample. It can be seen in the case of using a pulse shaper (Figure 3b), the assumption of the equality of forces at the ends is fulfilled more qualitatively.

The strain pulses recorded in the measuring bars were synchronized in time. According to the Kolsky formulas, for each experiment, the stress *σ*(t), strain *ε*(t), and strain rate $\dot{\varepsilon }$(t) as a function of time were plotted. The obtained dependences were synchronized in time for each strain rate condition. The time parameter having been excluded, a dynamic deformation diagram *σ*(*ε*) was constructed for the known strain rate history $\dot{\varepsilon }$(*ε*). Furthermore, for loading modes of the same type, averaged diagrams *σ*(*ε*) and $\dot{\varepsilon }$(*ε*) as well as the dependences of stresses on time *σ*(t) and strain rates on time $\dot{\varepsilon }$(t) were plotted. For each of the resulting diagrams, characteristic points with the maximum achieved stresses ($\sigma_{max}$) were identified. Those points correspond to moment after which the fracture of the samples occurred. For these points, the corresponding values of ultimate strains ($\varepsilon_{max}$) and time to failure ($\tau_{max}$) are determined. The strain rate ($\dot{\varepsilon }$) corresponding to the loading regime was taken to be the maximum value in the interval before the onset of fracture of the specimens.

The ultimate (maximum) stress reached at point A (Figure 3a) is the strength of the material. It should be noted that the process of loading from zero stress values to their maximum value proceeds at a variable strain rate both in the case of traditional loading without a pulse shaper in the transmitting bar and when a shaper is used. As shown by our studies with various materials (concrete, fiber-reinforced concrete, rocks), this effect occurs for all tested brittle materials. In this regard, when analyzing the influence of the strain rate, in some cases it is preferable to choose the value of the stress growth rate as a characteristic of the process of high-speed loading. As experience shows, it remains almost constant in active loading.

**Figure 4:**
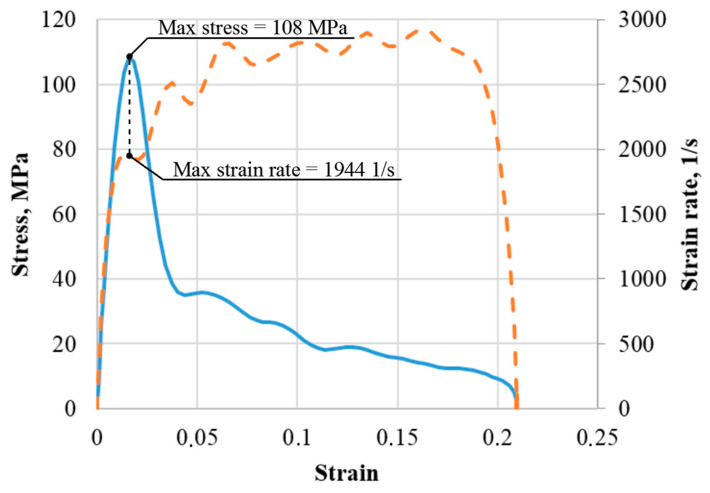
Strain rate characteristic of ultimate stress determination.

In the present study, the strain rate characteristic of ultimate stress was determined from the moment of the maximum stress achievement (the moment of failure) based on the history of stress and strain rate changes over time (Figure 4).

**Figure 5:**
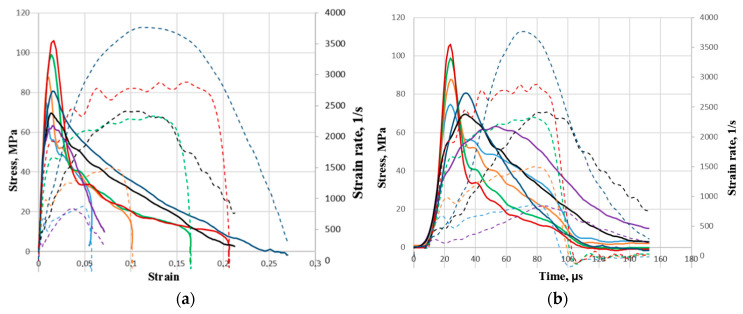
Average diagrams of dynamic deformation under compression: (**a**)—stress and strain rate dependencies on strain, (**b**)—time dependencies of stress and strain rate.

Figure 5 shows average strain diagrams over modes with a chronology of strain rate changes. Figure 5a shows the strain dependencies of the stress (solid lines and left vertical axis) and strain rate (dotted lines and right vertical axis), and the Figure 5b shows the corresponding time dependencies. The color represents the loading mode. In the above diagrams in the stress–strain axes *σ*(*ε*), the initial loading section is close to linear. With further deformation, when the ultimate stress values are reached, the concrete is intensively destroyed and is accompanied by a decrease in stresses and an increase in deformations. It can be seen that in the cases with and without a copper pulse shaper, the influence of the strain rate on the initial section of the diagrams is not observed under all loading modes. The magnitude of the maximum stresses is determined by the amplitudes of the incident pulses, that is, concrete samples during loading can withstand stresses that are significantly greater than its structural strength. This is due to the presence of two opposing processes: the rate of growth of longitudinal compressive loads, determined by the amplitudes of the loading waves, and the rates of formation and merging of microcracks during transverse expansion of the samples.

The dependences obtained demonstrate that with an increase in the strain rate, the maximum stresses increase. The ultimate strains corresponding to them also grow according to a linear law. The time before the onset of destruction decreases according to a power law. It can be seen that the use of pulse shapers reduced the range of strain rates and resulting stresses and increased the level of ultimate strains, and the process of loading the sample was extended in time, as was the destruction of the sample itself.

**Figure 6:**
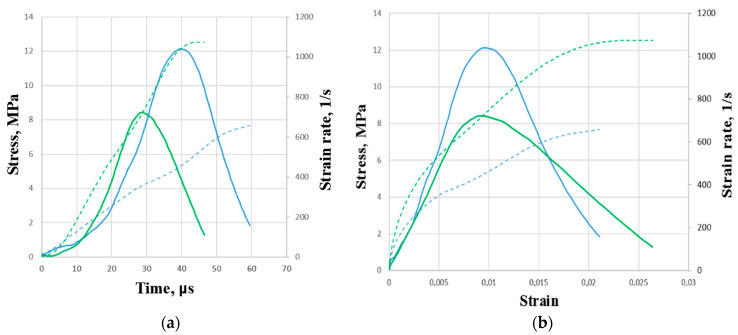
Averaged diagrams of the dependence of stress on strain and stress on time of modes No. 1 and No. 2 (**a**)—time dependencies of stress and strain rate, (**b**)—stress and strain rate dependencies on strain.

Direct tensile tests were carried out with two speed modes. In the first mode (Figure 6), there were five experimental shots; the average velocity of the impactor was 7 m/s, the average strain rate was about 500 reverse seconds, and the average maximum stress was 12 MPa. In Figure 6, the straight line shows the dependence on stress; the dashed line shows the dependence on the strain rate. In the second mode, there were also five experimental shots; the average velocity of the impactor was 11 m/s, the average strain rate was about 700 inverse seconds, and the average maximum stress was 8 MPa.

**Figure 7:**
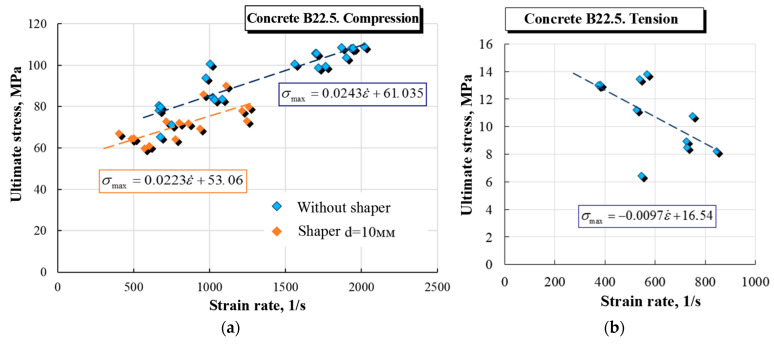
Rate dependences of ultimate stresses in compression (**a**) and tension (**b**).

The dynamic strength of concrete is characterized by the dynamic increase factor (DIF), which is obtained from the ratio of the maximum dynamic stress achieved in the experiment to the static compressive strength of concrete 22.5 MPa. The DIF value in the obtained strain rate range from ~400 to 2000 1/s varies from 1 to 5 (Figure 7). 

## 4. Identification of Computational Models

Explicit and implicit finite element analysis (FEA) offers a fast, reliable, and cost-effective solution for investigating the behavior of concrete and reinforced concrete structures under various load and strain rate conditions. Mathematical modeling is more profitable in contrast to time-consuming and expensive full-scale tests. However, when using the FEA approach, accurate material models are needed to describe the actual behavior of materials under various loading conditions. In case of impulse high-intensity loads, such as explosion and impact, FEA modeling of building structures evokes a serious problem.

The development of mathematical models of concrete behavior has been carried out for a long time [14,15,16,17,18], but due to the complex nonlinear behavior, a simple but accurate behavior model has not yet been formulated. Several complex models of concrete materials have been successfully used for dynamic analysis of concrete structures subjected to intense impact and explosive loads using the programs LS-DYNA and AUTODYN [19,20]. The number of required input parameters in these models ranges from 32 to 78.

At present, the materials library of the LS-DYNA software package contains about 250 models of materials of various physical natures [21], 31 of which are proposed to be used to describe the behavior of geomaterials, concretes, or rocks. In this work, the two most suitable models were selected from this list to describe the behavior of concrete: MAT_CONCRETE_DAMAGE_REL3 (No. 72) and MAT_CSCM (No. 159).

Model No. 72 (concrete damage) and its improved version MAT_CONCRETE_DAMAGE_REL3 [21,22] are used to simulate the behavior of concrete considering the fracture and the influence of the strain rate. This is a tri-invariant model that uses three fracture surfaces. Initially, it was based on the pseudotensor model. The most significant improvement for the user is the ability to generate model parameters based on the uniaxial compressive (uniaxial stress) strength of the concrete. The strength scaling factor is given by the curve for the entire range of strain rates. Tensile strain rates are defined by negative values. Numerical simulation results can be improved using custom input parameters that control the degradation of properties in compression and tension, the effect of strain rate, and the kinetics of damage accumulation.

Model No. 159 (CSCM) [23,24] uses a smooth “cap”-type flow surface. The strain rate is taken into account using the viscoplasticity model. The flow surface is formulated in terms of three stress tensor invariants: *j*’_1_ is the first stress tensor invariant, *j*’_2_ is the second stress tensor invariant, and *j*’_3_ is the third stress tensor invariant. These invariants are defined in terms of the deviator components of the stress tensor *S_ij_* and pressure *P*. At each calculation step, the viscoplasticity algorithm interpolates between the elastic trial stress *σ_ij_^T^* and the inviscid stress (without considering the strain rate) *σ_ij_^P^* to calculate the viscoplastic stress *σ_ij_^VP^*. The model makes it possible to take into account the influence of the strain rate on the strength characteristics of the material under tension, uniaxial compression, and shear. In addition, the user can take into account the influence of the strain rate on the fracture energy. To prevent an endless rise in DIF with strain rate, it is necessary to use an overstress intercept. For this purpose, Model 159 has two parameters: maximum overstress allowed in compression (OVERC) and maximum overstress allowed in tension (OVERT). In the present study, the maximum stresses were limited to the maximum stresses obtained in the experiments.

In modeling the process of dynamic deformation and destruction, the strain rate significantly affects the strength characteristics along with a significant difference of concrete properties in tension and compression. These effects were studied experimentally and then used to equip mathematical models with the necessary parameters.

The experimentally obtained strain rate dependences of strength are transferred to the model as a dependence of DIF on the strain rate. DIF was approximated by a power function:

$$\mathrm{DIF}\left(\dot{\varepsilon }\right)=1+k\cdot {\dot{\varepsilon }}^{n}$$

**Figure 8:**
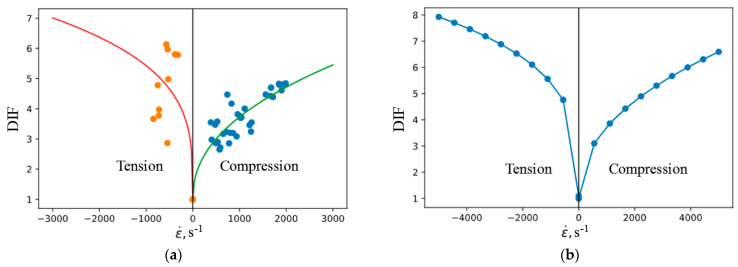
Rate dependences of ultimate stresses in compression and tension: (**a**) is an approximation of experimental data; (**b**) is a tabular dependence.

The parameters of approximations of DIF velocity are presented in Table 3. A comparison of data with approximating functions is shown in Figure 8a.

In the MAT_72 model, the speed dependence DIF is given by a table function. Negative strain rates correspond to tension. The value of DIF at a strain rate equal to 0 should be equal to 1. To obtain such a function, 20 points were uniformly selected in the range of strain rates from −5000 1/s to 5000 1/s. The DIF values at the corresponding points were calculated using the approximating dependences of the experimental data. The obtained data are presented in Figure 8b.

There are two modes of using Model 159: automatic generation of model parameters for concrete specified by static compressive strength (*fc*) and manual input of model parameters. In the first case, it is impossible to correct part of the model parameters in order to refine the behavior of a particular material. In the second case, it is necessary to set the values of 37 material constants. The work [23] describes the algorithms for calculating the parameters that are used by default. These algorithms are based on the interpolation of the values of the corresponding material parameters according to the data obtained for concretes with different static compressive strength. The parameters for concrete B22.5, independent of the strain rate and calculated in accordance with the dependencies embedded in the model, are presented in Table 4.

To take into account the influence of the strain rate on the ultimate stresses of concrete, the model provides the following six parameters:

- *η*0*c* and *ηc*—for compression;
- *η*0*t* and *ηt*—for tensile;
- *η*0*s* and *ηs*—for shear.

The dynamic limit stress is calculated using the formulas:

$$f_{c}^{dyn}=f_{c}+E\cdot \dot{\varepsilon }\cdot \frac{\eta 0c}{{\dot{\varepsilon }}^{\eta c}}$$

$$f_{t}^{dyn}=f_{t}+E\cdot \dot{\varepsilon }\cdot \frac{\eta 0t}{{\dot{\varepsilon }}^{\eta t}}$$

$$f_{c}^{dyn}=f_{s}+E\cdot \dot{\varepsilon }\cdot \frac{\eta 0s}{{\dot{\varepsilon }}^{\eta s}}$$

where the effective strain rate $\dot{\varepsilon }$ is determined by the expression:

$$\dot{\varepsilon }=\sqrt{\frac{2}{3}[{\left({\dot{\varepsilon }}_{x}-{\dot{\varepsilon }}_{y}\right)}^{2}+{\left({\dot{\varepsilon }}_{x}-{\dot{\varepsilon }}_{z}\right)}^{2}+{\left({\dot{\varepsilon }}_{z}-{\dot{\varepsilon }}_{y}\right)}^{2}+{{\dot{\varepsilon }}_{x}}^{2}+{{\dot{\varepsilon }}_{x}}^{2}+{{\dot{\varepsilon }}_{z}}^{2}]}$$

**Figure 9:**
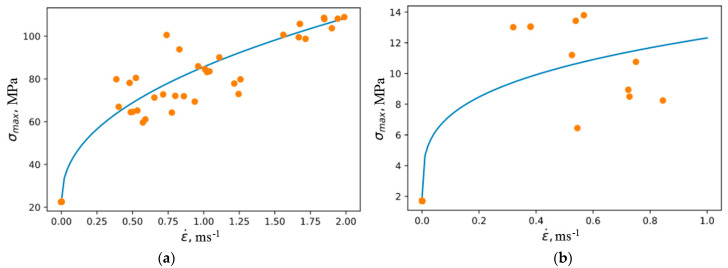
Approximation of the rate dependences of the ultimate stress in compression (**a**) and tension (**b**).

To determine these parameters, the previously obtained experimental velocity dependences of the ultimate stresses in compression and tension were approximated as follows, see Figure 9. Parameter values are given in Table 5.

Model No. 159 includes the possibility of taking into account the influence of the strain rate on the fracture energy of the material (separately in compression, tension, and shear). These dependencies are determined by the formulas:

$$G_{fc}^{dyn}=G_{fc}\cdot {\left(1+\frac{E\cdot \dot{\varepsilon }}{f_{c}}\cdot \frac{\eta 0c}{{\dot{\varepsilon }}^{\eta c}}\right)}^{repow}$$

$$G_{ft}^{dyn}=G_{ft}\cdot {\left(1+\frac{E\cdot \dot{\varepsilon }}{f_{t}}\cdot \frac{\eta 0t}{{\dot{\varepsilon }}^{\eta t}}\right)}^{repow}$$

To determine the values of the parameters *G_fc_*, *G_ft_*, and *repow* for each test, the fracture energy was determined. This energy corresponds to the area under the strain diagram of the sample beyond the point of maximum stress that leads to fractures.

**Figure 10:**
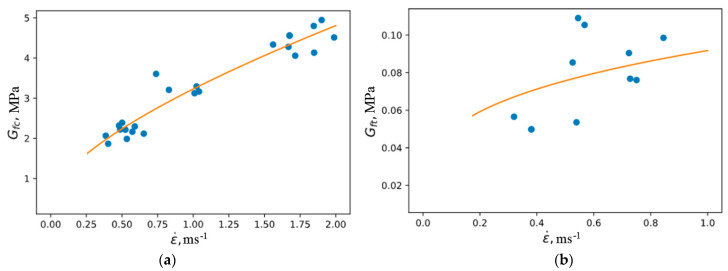
Approximation of the rate dependence of the fracture energy in compression (**a**) and tension (**b**).

The results of the approximation of the rate dependences of the fracture energy in compression and tension are shown in Figure 10. The parameters are given in Table 6.

Thus, a complete set of parameters has been defined to describe the dynamic deformation and fracture of B22.5 concrete within the framework of the MAT_159 model.

**Figure 11:**
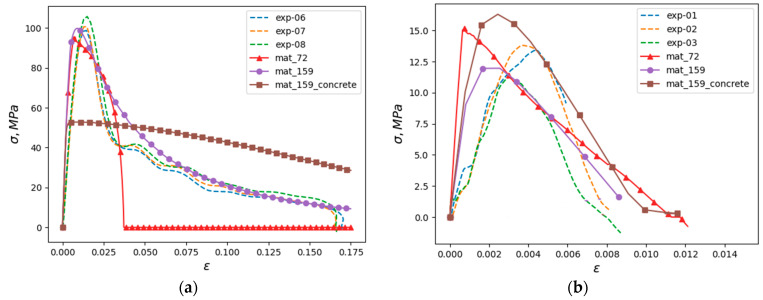
Comparison of stresses in an elementary volume: experiment and prediction using different models (**a**) compression, (**b**) tension.

Stress–strain diagrams obtained during compression and tension of an elementary volume at a constant strain rate are compared in different models. In simulation, one hexahedral element was loaded under one-dimensional stress conditions. Symmetry conditions were set on three faces of the element. Two faces were free and normal displacement was predefined of one face to obtain a constant specified strain rate. The results are shown in Figure 11. The dotted line shows the results of the real-world tests; the mat_52 legend corresponds to the calculation with the MAT_72 material, and the mat_159 legend corresponds to the calculation with the MAT_159 model, in which the main part of the parameters was determined from the dependencies embedded in the model and the parameters characterizing the strain rate effects were determined from the experimental data. The mat_159_concrete curve was obtained using a model in which all parameters, including those responsible for strain rate effects, were calculated according to the algorithms embedded in the model for a given concrete strength class. It should be noted that for tension, the slope of the initial section of the diagrams in the experiment and calculation are very different, which is apparently due to the error in measuring deformations using measuring bars. The strains realized under tension are an order of magnitude smaller than the strains under compression.

It can be noted that the fracture branch of the curves is qualitatively best described by Model 159, in which some parameters were set manually. Apparently, this is due to the fact that in this case, the strain rate dependence of the fracture energy of the material was explicitly specified. The model automatically generated from the static strength of the material (line with square markers) predicts a significantly lower maximum stress and correlates poorly with the experimental data beyond the fracture initiation point.

## 5. Conclusions

As a result of the experimental study, the strain rate dependences of the ultimate stresses of concrete—as well as the fracture energy—under compression and tension were obtained. The ultimate stresses during compression and tension are affected by the strain rate. The dynamic strength values differ from static values by six times in the studied range. Based on the series of experiments on concrete samples, the identification was carried out, and the parameters for the MAT_CONCRETE_DAMAGE_REL3 (No. 72) and MAT_CSCM (No. 159) models were determined taking into account the effect of strain rate on strength characteristics as well as a significant difference in the properties of concrete in tension and compression. As a result of testing the models by the single-element modeling method, it was shown that the verified models qualitatively and quantitatively, with an accuracy acceptable for practical needs, make it possible to predict the results of real-world tests.

## Figures and Tables

**Table 1:** Parameters of experiments for uniaxial compression.

Mode	Experiment Code	Stricker Velocity, m/s	Max Stress, MPa	Ultimate Strain, %	Strain Rate, 1/s	Lifetime, ms	DIF	Specific Fracture Energy, MPa
1	c637-19	14.13	78.14	0.89	665.74	25.00	3.47	2.70
	c637-20	13.67	80.49	0.80	666.23	17.50	3.58	2.51
	c637-21	13.44	65.24	0.83	676.59	21.25	2.90	2.30
	c637-22	13.62	79.86	0.79	676.59	21.25	3.55	2.38
	c637-23	14.2	71.28	0.83	748.59	20.00	3.17	2.47
2	c637-01	18.02	84.55	0.93	1019.51	16.25	3.76	3.61
	c637-02	18.33	83.21	0.98	1032.09	17.50	3.70	3.51
	c637-03	18.62	93.81	1.08	974.41	17.50	4.17	3.79
	c637-04	18.68	83.54	1.10	1084.05	17.50	3.71	3.70
	c637-05	18.09	100.56	1.12	1003.36	17.50	4.47	4.02
3	c637-06	25.86	98.75	1.46	1717.47	17.50	4.39	5.06
	c637-07	25.77	100.64	1.29	1560.29	17.50	4.47	5.15
	c637-08	26.45	105.76	1.50	1698.34	17.50	4.70	5.62
	c637-09	26.2	99.50	1.32	1764.55	18.75	4.42	5.16
	c637-10	25.75	105.75	1.47	1704.93	18.75	4.70	5.57
4	c637-11	31.05	108.61	1.65	1870.60	15.00	4.83	6.03
	c637-12	30.72	108.94	1.44	2019.84	13.75	4.84	5.51
	c637-13	31.11	103.71	1.46	1900.78	13.75	4.61	5.97
	c637-14	30.73	108.00	1.80	1931.89	16.25	4.80	5.47
	c637-15	31.45	108.20	1.59	1944.06	13.75	4.81	6.29
5	c637-24	18.4	66.96	1.21	403.13	50.00	2.98	2.34
	c637-25	19.09	60.98	1.81	604.31	52.50	2.71	2.97
	c637-26	18.46	59.65	1.72	572.70	52.50	2.65	2.83
	c637-27	18.55	64.62	1.53	501.15	47.50	2.87	2.94
6	c637-28	18.6	64.45	1.51	487.37	51.25	2.86	2.77
	c637-30	35.25	71.92	1.51	860.89	30.00	3.20	6.51
	c637-31	35.65	72.07	1.30	800.43	28.75	3.20	6.43
	c637-32	35.76	72.76	1.24	714.93	26.25	3.23	6.35
	c637-33	35.63	69.39	1.62	937.20	32.50	3.08	6.64
	c637-34	35.63	64.27	1.32	776.42	28.75	2.86	5.78
7	c637-46	60.98	90.10	1.68	1109.74	28.75	4.00	8.07
	c637-47	60.27	85.94	1.33	961.21	21.25	3.82	7.97
	c637-48	60.73	79.81	1.63	1258.44	25.00	3.55	8.46
	c637-49	59.77	77.84	1.63	1215.06	27.50	3.46	7.42
	c637-50	61.15	72.97	1.69	1245.61	26.25	3.24	7.19

**Table 2:** Parameters of direct tensile experiments.

Mode	Experiment Code	Stricker Velocity, m/s	Max Stress, MPa	Ultimate Strain, %	Strain Rate, 1/s	Lifetime, ms	DIF	Specific Fracture Energy, Mpa
1	t637-01	7.11	13.43	1.31	539.07	43	5.97	0.054
	t637-02	7.87	13.79	1.1	567.69	34.5	6.13	0.108
	t637-03	7.64	11.2	0.89	525.76	35	4.98	0.088
	t637-04	6.38	13.04	0.831	380.64	34	5.80	0.052
	t637-05	5.94	13.01	1	373.56	39	5.78	0.058
2	t637-06	10.85	8.94	0.92	723.52	25.5	3.97	0.093
	t637-07	11.23	8.24	1.11	844.88	26.5	3.66	0.100
	t637-08	11.39	10.75	0.95	750.21	21	4.78	0.080
	t637-09	10.54	6.44	0.66	545	24	2.86	0.110
	t637-10	10.95	8.49	0.93	727.69	29.5	3.78	0.079

**Table 3:** Approximation parameters for the speed dependences of the strength of concrete class B22.5.

	*k*	*n*
Compression	0.125	0.446
Tension	0.65	0.28

**Table 4:** B22.5 concrete model parameters.

**Modulus of elasticity**	*E* [GPa]	23.947	**Parameters defining the triaxial expansion surface**	*α*_2_ [-]	0.66
	*K* [GPa]	11.4		*λ*_2_ [-]	0.16
	*G* [GPa]	10.4		*β*_2_ [MPa^−1^]	0.077
	*ν* [-]	0.15		*θ*_2_ [MPa^−1^]	0.0016
**Static tensile strength**	*f_t_* [MPa]	1.79			
**Parameters defining the surface of triaxial compression**	*α* [MPa]	13.3	**Parameters of the “cap” surface**	*R* [-]	5
	*λ* [MPa]	10.5		*X*_0_ [MPa]	87.8
	*β* [MPa^−1^]	0.01929	**Porosity of concrete**	*W* [-]	0.05
	*θ* [-]	0.27	**Parameters that determine hardening on the “pressure-volume deformation” curve**	*D*_1_ [MPa]	*2.5 × 10^−4^*
**Parameters defining the surface of torsion**	*α_1_* [-]	0.747		*D*_2_ [MPa^2^]	*3.49 × 10^−7^*
	*λ*_1_ [-]	0.17			
	*β*_1_ [MPa^−1^]	0.0766			
	*θ*_1_ [MPa^−1^]	0.0013			

**Table 5:** Parameters that determine the effect of strain rate on ultimate stresses.

***η*0*c* [-]**	***ηc* [-]**	***η*0*t* [-]**	***ηt* [-]**
2.6 × 10^−3^	0.55	4.4 × 10^−4^	0.72

**Table 6:** Parameters of rate dependences of the fracture energy.

*G_fc_* [MPa]	*G_ft_* [MPa]	*Repow* [-]
0.341	3.5·10^−3^	1.681

## Data Availability

The data presented in this study are available on request from the corresponding author.
